# Effectiveness of Adalimumab for the Treatment of Psoriatic Arthritis: An Italian Real-Life Retrospective Study

**DOI:** 10.3389/fphar.2019.01497

**Published:** 2019-12-13

**Authors:** Salvatore D'Angelo, Fabrizio Cantini, Roberta Ramonda, Luca Cantarini, Antonio Carletto, Maria Sole Chimenti, Andrea Delle Sedie, Rosario Foti, Roberto Gerli, Claudia Lomater, Ennio Lubrano, Antonio Marchesoni, Alen Zabotti, Carlo Salvarani, Rossana Scrivo, Raffaele Scarpa, Giuseppina Tramontano, Carlotta Nannini, Mariagrazia Lorenzin, Marta Fabbroni, Federica Martinis, Roberto Perricone, Linda Carli, Elisa Visalli, Guido Rovera, Fabio Massimo Perrotta, Luca Quartuccio, Alessio Altobelli, Luisa Costa, Laura Niccoli, Augusta Ortolan, Francesco Caso

**Affiliations:** ^1^Rheumatology Department of Lucania, Rheumatology Institute of Lucania (IReL), Potenza, Italy; ^2^Basilicata Ricerca Biomedica (BRB), Potenza, Italy; ^3^Department of Rheumatology, Hospital of Prato, Prato, Italy; ^4^Rheumatology Unit, Department of Medicine-DIMED, University of Padova, Padova, Italy; ^5^Research Center of Systemic Autoinflammatory Diseases, Behçet's Disease Clinic and Rheumatology-Ophthalmology Collaborative Uveitis Center, Department of Medical Sciences, Surgery and Neurosciences, University of Siena, Siena, Italy; ^6^Rheumatology Unit, University of Verona, Verona, Italy; ^7^Rheumatology, Allergology and Clinical Immunology, Department of Medicina dei Sistemi, University of Rome Tor Vergata, Rome, Italy; ^8^Rheumatology Unit, Department of Clinical and Experimental Medicine, University of Pisa, Pisa, Italy; ^9^Rheumatology Unit, Vittorio-Emanuele University Hospital of Catania, Catania, Italy; ^10^Rheumatology Unit, Department of Medicine, University of Perugia, Perugia, Italy; ^11^Rheumatology Unit, Ospedale Mauriziano, Torino, Italy; ^12^Academic Rheumatology Unit, Dipartimento di Medicina e Scienze della Salute, Università degli studi del Molise, Campobasso, Italy; ^13^Department of Rheumatology, ASST Centro Specialistico Ortopedico Traumatologico Gaetano Pini-CTO, Milan, Italy; ^14^Rheumatology Clinic, Department of Medical Area, University of Udine, Academic Hospital S. Maria della Misericordia, Udine, Italy; ^15^Rheumatology Unit, Deptartment of Internal Medicine, Azienda USL-IRCCS, Istituto di Ricovero e Cura a Carattere Scientifico, Reggio Emilia, Italy; ^16^University of Modena and Reggio Emilia, Modena, Italy; ^17^Dipartimento di Medicina Interna e Specialità Mediche, Reumatologia, Sapienza Università di Roma, Rome, Italy; ^18^Rheumatology Unit, Department of Clinical Medicine and Surgery, School of Medicine and Surgery, University of Naples Federico II, Naples, Italy

**Keywords:** psoriatic arthritis, biological drugs, adalimumab, retention rate, real-life

## Abstract

**Background:** Few studies have evaluated the effectiveness of adalimumab in the real-life setting in psoriatic arthritis (PsA).

**Objective:** To evaluate the 2-year retention rate of adalimumab in PsA patients. Potential baseline parameters influencing persistence on treatment were also evaluated.

**Methods:** PsA patients from 16 Italian Rheumatology Units treated with adalimumab as first- or second-line biological therapy were retrospectively evaluated. Adalimumab retention rate was evaluated at 12 and 24 months. Logistic regression was used to evaluate the association between predictor variables and adalimumab retention rate.

**Results:** From 424 patients (53.5% male, aged 48.3 ± 12.8 years) who started treatment with adalimumab, 367 (86.6%) maintained treatment for 12 months and 313 (73.8%) for 2 years. At 24-months, Disease Activity in PsA (DAPSA) remission (defined as ≤4) and Low Disease Activity (LDA) (≤14) were achieved in 22.8% and 44.4% of patients, respectively. Adalimumab treatment significantly decreased the number of tender (7.0 ± 5.7 at baseline vs. 2.3 ± 3.5 at 24 months, p < 0.001) and swollen joints (2.7 ± 2.8 at baseline vs. 0.4 ± 0.9 at 24 months, p < 0.001), DAPSA (25.5 ± 10.9 at baseline vs. 11.0 ± 8.4 at 24 months, p < 0.001), PASI (5.3 ± 5.7 at baseline vs. 2.7 ± 2.8 at 24 months, p < 0.001) and CRP (3.8 ± 6.3 at baseline vs. 1.2 ± 1.7 at 24 months, p < 0.001). Among a range of laboratory and clinical variables, only female gender was associated with improved adalimumab persistence at 24 months (OR: 1.98, 95% CI: 1.2–3.2, p = 0.005).

**Conclusions:** Independent of a range of predictor variables, adalimumab was shown to be effective, while maintaining a high retention rate after 2 years in PsA patients.

## Introduction

Psoriatic arthritis (PsA) is a chronic and invalidating disease characterized by joint and entheseal inflammation affecting 0.05–0.25% of the general population and 6–41% of patients with psoriasis (Gottlieb and Dann, 2009; Laws et al., 2010; Olivieri et al., 2014; Ogdie and Weiss, 2015).

Up until two decades ago, treatment of PsA was often unsatisfactory. Findings based on the immunopathogenesis of the disease have led to the development of biological drugs directed against specific (pathogenetic) targets, in particular tumor necrosis factor-α (TNFα). TNFα is a pleiotropic cytokine which regulates several inflammatory reactions and immune functions through the control of cellular processes and plays a central role in the pathogenesis of PsA (Mantravadi et al., 2017). Anti-TNFα drugs have opened new therapeutic horizons in PsA, proving to be effective in the control of the signs/symptoms of inflammation, in improving the quality of life and the functional outcome, in inhibiting the progression of the structural damage in the peripheral joints and presenting a good safety profile (D'Angelo et al., 2012; Perrotta and Lubrano, 2016; D'Angelo et al., 2017). Treatment strategies of active, predominantly peripheral PsA recommended by International and National Guidelines suggest to use conventional disease-modifying drugs anti-rheumatic (DMARDs), such as methotrexate (MTX). In cases of inadequate response, contraindication or intolerance to at least one DMARD, treatment with biological drugs such as TNFα (adalimumab, infliximab, etanercept, golimumab, or certolizumab pegol) or anti-interleukin therapies (ustekinumab or secukinumab) should be considered (Gossec et al., 2016; Marchesoni et al., 2017).

Adalimumab has been shown to be effective and reasonably safe in reducing disease activity and controlling joint damage in patients with PsA, even in comorbid conditions (D'Angelo et al., 2012). However, despite its generally high efficacy, some patients with PsA may be refractory to adalimumab therapy, may lose response or develop drug intolerance over time (Perrotta and Lubrano, 2016; D'Angelo et al., 2017). The persistence in therapy in real-life clinical practice is increasingly recognised as a surrogate marker for the efficacy and safety of a drug (Saad et al., 2011). National registries provide clinical data from the real-world setting, with the aim to monitor long-term safety of a specific treatment, but they also yield other important information (difficult to achieve in clinical trials), such as drug survival and long-term effectiveness (Armuzzi et al., 2014).

The present real-life study evaluated the persistence of adalimumab in the management of PsA patients over a period of 2 years. Potential baseline clinical and laboratory parameters influencing persistence rate were also evaluated.

## Methods

### Patients and Study Design

The present retrospective non-interventional longitudinal study included consecutive PsA patients who started a treatment with adalimumab as of 1^st^ January 2013 in 16 Italian Rheumatology Centres. Inclusion criteria were the following: age ≥18 years; diagnosed with active PsA and having started a treatment with adalimumab in routine clinical practice, regardless of whether they were biologic naïve or whether they had previously received biologic treatment. Active PsA was defined by a rheumatologist based on clinical judgment considered peripheral arthritis, enthesitis or axial involvement. Diagnosis of PsA was clinical (D'Angelo et al., 2016) and in addition, all patients satisfied CASPAR (ClASsification criteria for Psoriatic ARthritis) criteria for the classification of PsA (Taylor et al., 2006). Patients' written consent were obtained according to the Declaration of Helsinki when patients were first entered into the database for treatment. Ethics committee approval from all participating centres and written informed consent for the anonymous use of personal data were obtained from every patient, in compliance with the Italian Legislative Decree 196/2003.

All participating Centres have a recognised expertise in the management of PsA and regularly collect data using a standardized database on the efficacy and safety of patients with PsA treated with biological drugs. For the purpose of this study, the data extracted from the database were the following: demographic features (age, sex, and time since PsA diagnosis), clinical parameters (tender and swollen joints, dactylitis, enthesitis assessed by physical examination according to the expanded Leeds index, psoriasis according to Psoriasis Area Severity Index [PASI], Disease Activity index for Psoriatic Arthritis [DAPSA]) and treatment (previous biologics, previous conventional DMARD, combined treatment, dose of adalimumab) at the time of initiation and during the follow-up of adalimumab treatment. The analysis was performed on data at three time-points: baseline, 12 and 24 months.

### Outcome Measures

Drug retention was retrospectively evaluated as the number of patients (%) on treatment until definitive treatment interruption over the study period. Reasons for discontinuation were analysed and classified into the following categories: 1) lack of effectiveness (including primary and secondary); 2) adverse events (infection, skin or systemic reaction, and other adverse events, including hematologic, pulmonary, renal, cardiovascular complications, and malignancies, etc.); and 3) other reasons (patient preference, change in hospital, desire for pregnancy, disease remission, etc.). The effectiveness of adalimumab treatment was also evaluated at 24 months and was defined as the proportion of patients achieving remission, defined as a DAPSA score ≤4 and low disease activity (LDA) as >4 and ≤14 (Gossec et al., 2016 and Schoels et al., 2016) .The effect of adalimumab treatment on a range of clinical and laboratory features and disease activity variables (tender and swollen joint count, CRP, dactylitis, enthesitis, PASI and DAPSA) and extra-articular manifestations (Crohn's disease, uveitis) was also evaluated at 12 and 24 months.

### Statistical Analysis

No formal power calculation was performed since this was a retrospective longitudinal study that included consecutive PsA patients seen in a real-life setting. Data are presented as mean ± SD or number and %. Comparisons in variables between two groups (i.e. patients discontinuing vs. those continuing adalimumab treatment at 24 months) were performed by univariate analysis using the Chi-squared test for categorical variables or the Mann-Whitney U- Test for non-parametric continuous variables. Three groups were compared (i.e. baseline, 12 and 24 months) by 1-way ANOVA followed by Bonferroni post-hoc test to account for α-inflation by type-1 error derived from multiple testing. Variables that were found to be statistically significant predictors following univariate analysis were included in multivariate regression models. A p-value of ≤0.05 was considered statistically significant. Statistical analyses were performed using SPSS statistical software, version 20.0 (SPSS, Chicago, IL, USA).

## Results

### Baseline Clinical Characteristics

A total of 424 PsA patients were included in the present study. Baseline clinical characteristics are summarised in **Table 1**. The majority of patients were male (N = 227, 53.5%) and mean age was 48.3 ± 12.8 years. Three hundred and fifteen (74.3%) patients had peripheral arthritis, 148 (47.3%) enthesitis, 87 (27.8%) dactylitis, 81 (19.1%) axial involvement and 306 (72.5%) had concomitant psoriasis. Extra-articular complications such as uveitis (N = 27, 6.4%) and Crohn's disease (N = 23, 5.4%) were less frequent. Frequent comorbid diseases at baseline included hypertension (N = 130, 30.8%) and metabolic syndrome (N = 75, 17.9%). The majority of patients presented with moderately active disease, as observed by DAPSA score (25.5 ± 10.9). Prior to undertaking treatment with adalimumab, 291 (68.6%) patients were biologic naïve while almost all patients received conventional DMARDs (N = 404, 95.3%). As regards the 133 patients with a previous biologic treatment, 33 had discontinued due to primary inefficacy, 64 due to secondary inefficacy, 27 due to adverse events and 9 due to other reasons.

**Table 1:** Baseline clinical characteristics of psoriatic arthritis patients.

Clinical characteristics	PsA (N = 424)
*General*	
Male patients, n (%)	227 (53.5)
Age (years)	48.3 ± 12.8
BMI (Kg/M^2^)	25.8 ± 4.4
Current smoker, n (%)	84 (19.8)
Disease duration from arthritis onset (years)	7.6 ± 7.2
*Arthritis assessment*	
Arthritis, n (%)	315 (74.3)
Tender joint count	7.0 ± 5.7
Swollen joint count	2.7 ± 2.8
CRP (mg/dl)	3.8 ± 6.3
DAPSA	25.5 ± 10.9
Enthesitis, n (%)	148 (47.3)
Dactylitis, n (%)	87 (27.8)
Spondylitis, n (%)	81 (19.1)
*Skin assessment*	
Current psoriasis, n (%)	306 (72.5)
PASI score	5.3 ± 5.7
Comorbidities, n (%)*	
Hypertension	130 (30.8)
Metabolic syndrome	75 (17.9)
Depression	38 (9.2)
Hyperuricemia	32 (7.7)
Cardiovascular disease	28 (6.7)
Uveitis	27 (6.4)
Crohn's disease	23 (5.4)
Ulcerative colitis	12 (2.8)
*Medication, n (%)*	
DMARD	404 (95.3)
Methotrexate	321 (75.7)
Biologic naïve	291 (68.6)
Adalimumab	
Monotherapy	190 (44.8)
plus methotrexate	183 (43.2)
plus other DMARD	51 (12.0)

Data are presented as mean ± SD or number (%). BMI, body mass index; CRP, C-reactive protein; DAPSA, disease activity in psoriatic arthritis; DMARD, disease-modifying anti-rheumatic drugs; PASI, psoriasis area severity index; PsA, psoriatic arthritis.*including extra-articular manifestations.

### Adalimumab Treatment

Adalimumab was administered as monotherapy in 190 patients (44.8%) and combined with MTX in 183 (43.2%) patients, the majority (N = 164, 89.6%) receiving MTX 10-20 mg (mean dose of 13.1 ± 3.0 mg) per week. Adalimumab was administered in combination with DMARDs other than MTX including sulfasalazine, leflunomide, cyclosporine and hydroxychloroquine in 51 (12%) patients.

### Effectiveness

**Figure 1:**
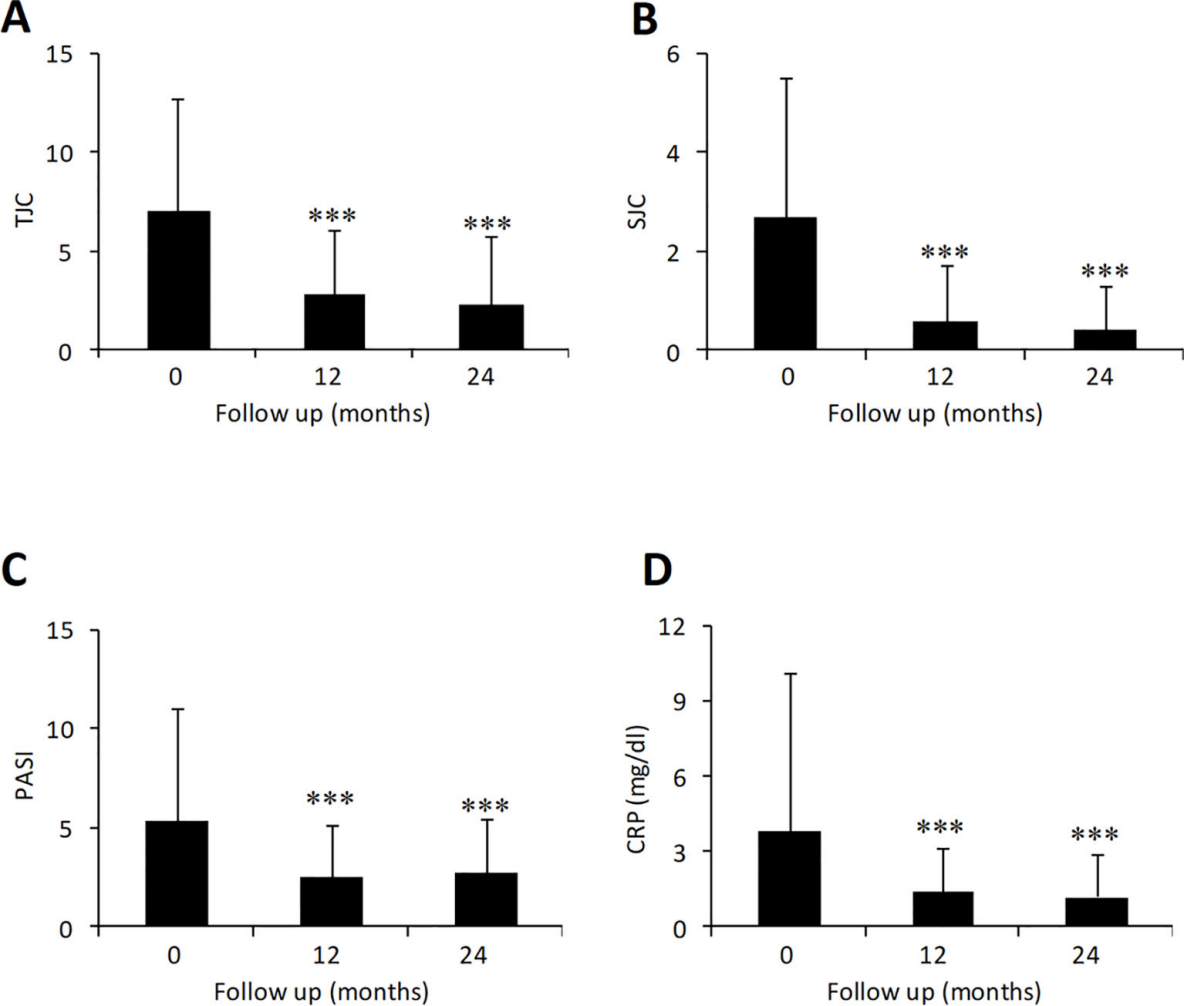
Effect of adalimumab treatment in PsA patients on tender [**(A)** N = 303, 271, 275 at 0, 12, and 24 months] and swollen joints [**(B)** N = 303, 271, 275 at 0, 12, and 24 months], CRP [**(C)** N = 298, 269, 272 at 0, 12, and 24 months] and PASI score [**(D)** N = 99, 73, 39 at 0, 12, and 24 months]. CRP, C-reactive protein; PASI, psoriasis area severity index; SJC, swollen joint count; TJC, tender joint count. Data presented as mean ± SD. Asterisks denote statistically significant differences compared to baseline values after 1-way ANOVA followed by Bonferroni post-hoc test.

**Figure 2:**
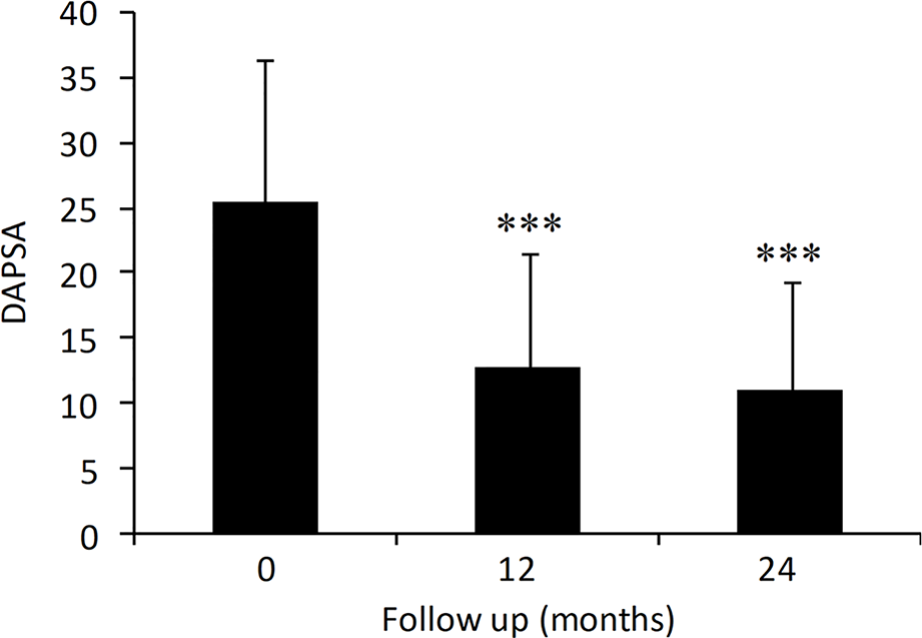
Effect of adalimumab treatment in PsA patients on DAPSA (N = 209, 183, 189 at 0, 12 and 24 months). DAPSA, disease activity in psoriatic arthritis. Data are presented as mean ± SD. Asterisks denote statistically significant differences compared to baseline values after 1-way ANOVA followed by Bonferroni post-hoc test.

The effect of adalimumab treatment was evaluated on some clinical and laboratory measures (**Figure 1**). Both tender joint count (TJC) and swollen joint count (SJC) were significantly decreased compared to baseline values (TJC: 7.0 ± 5.7 at baseline vs. 2.3 ± 3.5 at 24 months, p < 0.001; SJC: 2.7 ± 2.8 at baseline vs. 0.4 ± 0.9 at 24 months, p < 0.001) (**Figures 1A**, **B**). Similarly, PASI (5.3 ± 5.7 at baseline vs. 2.7 ± 2.8 at 24 months, p < 0.001) and CRP (3.8 ± 6.3 at baseline vs. 1.2 ± 1.7 at 24 months, p < 0.001) were significantly decreased in patients treated with adalimumab over the 2-year period (**Figures 1C**, **D**). Mean DAPSA score was significantly decreased compared to baseline values after 12 and 24 months (25.5 ± 10.9 at baseline vs. 11.0 ± 8.4 at 24 months, p < 0.001) of adalimumab treatment (**Figure 2**). Clinical remission and LDA at 24 months were achieved in 22.8% and 44.4% of patients, respectively.

In addition, patients with enthesitis (47.3% at baseline vs. 15.1% at 24 months, p < 0.001) and dactylitis (27.8% at baseline vs. 3.2% at 24 months, p < 0.001) were also significantly decreased over the follow-up period.

### Adalimumab Retention Rate and Factors Influencing Retention at 24 Months

Of the 424 patients who started treatment with adalimumab, 367 (86.6%) maintained treatment for 12 months and 313 (73.8%) for 24 months.

Univariate and multivariate analyses were used to examine predictors of 24-month adalimumab persistence in PsA patients. Considering all potential variables compared using univariate analyses (**Table 2**), only high baseline CRP levels (3.2 ± 5.3 mg/dl discontinuing vs. 3.8 ± 6.3 mg/dl in adalimumab continuing patients, p = 0.047) and female gender (34.2% in discontinuing vs. 50.8% in adalimumab continuing patients, p = 0.004) emerged as being significantly associated with improved adalimumab persistence at 24 months. Stratification of patients based on concomitant treatment (e.g. adalimumab monotherapy vs. adalimumab plus MTX or adalimumab plus other DMARD) did not reveal any significant difference in adalimumab retention (p = 0.46) (**Table 2**). Furthermore, the presence of comorbid diseases or exposure to previous biologic were not associated with adalimumab retention. In a multivariate regression model (including only gender and high baseline CRP levels), only female sex emerged as a significant predictor of improved adalimumab retention at 24 months (OR 1.98, 95% CI 1.2–3.2, p = 0.005).

**Table 2:** Predictor variables of adalimumab persistence at 24 months.

Clinical characteristics	Adalimumab treatment		P-value
	Discontinuing (N = 111)	Continuing (N = 313)	
*General*			
Female patients, n (%)	38 (34.2)	159 (50.8)	**0.004**
Age (years)	47.9 ± 13.1	48.4 ± 12.7	0.69
BMI (Kg/M^2^)	25.4 ± 4.5	25.9 ± 4.3	0.19
Current smoker, n (%)	24 (27)	60 (20.5)	0.26
*Arthritis assessment*			
Tender joint count	7.3 ± 6	7 ± 5.7	0.93
Swollen joint count	2.6 ± 2.8	2.7 ± 2.8	0.71
CRP (mg/dl)	3.2 ± 5.3	3.8 ± 6.3	**0.047**
DAPSA	24.7 ± 9.8	25.5 ± 10.9	0.61
Enthesitis	54 (48.6)	148 (47.3)	0.89
Dactylitis	21 (19.1)	87 (27.8)	0.09
*Skin assessment*			
PASI	4.4 ± 4.3	5.3 ± 5.7	0.63
Comorbidities*, n (%)			
Hypertension	31 (28.2)	99 (31.7)	0.57
Metabolic syndrome	19 (17.6)	56 (17.9)	1.0
Depression	12 (11.1)	26 (8.5)	0.53
Hyperuricemia	4 (3.7)	28 (9.1)	0.12
Cardiovascular disease	7 (6.5)	21 (6.8)	1.0
Uveitis	7 (6.4)	20 (6.4)	1.0
Crohn's disease	4 (3.6)	19 (6.1)	0.47
Ulcerative colitis	3 (2.7)	9 (2.9)	1.0
*Medication, n (%)*			
Biologic naïve	74 (66.7)	217 (69.3)	0.69
Adalimumab			
Monotherapy	48 (43.2)	142 (45.4)	0.46
Plus methotrexate	46 (41.4)	137 (43.8)
Plus other DMARD	17 (15.3)	34 (10.9)

Data are presented as mean ± SD or number (%). N refers to number of patients treated with adalimumab for 24 months. BMI, body mass index; CRP, C-reactive protein; DAPSA, disease activity in psoriatic arthritis; DMARD, disease-modifying anti-rheumatic drugs; PASI, psoriasis area severity index; PsA, psoriatic arthritis.*including extra-articular manifestations. Comparisons in variables between patients discontinuing vs. those continuing adalimumab treatment at 24 months were performed using the Chi-squared test for categorical variables or the Mann-Whitney U-Test for non-parametric continuous variables. P-values reporting statistically significant differences are highlighted in bold.

### Reasons for Adalimumab Discontinuation

Over the 24-month treatment period, adalimumab was suspended in a total of 111/424 (26.2%) patients. Reasons for discontinuation were primary inefficacy (N = 30, 7.1%), secondary inefficacy (N = 15, 3.5%), adverse events (N = 26, 6.1%; subjective intolerance, allergic reaction, biliary colic, diplopia, and paresthesia in limbs or other side effects) and other reasons (N = 40, 9.4%; lost during follow up, pregnancy, paternity leave, not reported, or not recorded).

## Discussion

Efficacy and safety data currently available for anti-TNFα drugs for the treatment of PsA are mainly derived from randomised clinical trials (RCTs). Although RCTs still represent the most powerful research tool to confirm the efficacy of a treatment, results emerging from these studies are based on a selected population, with the exclusion of co-morbidities, treated and observed for a limited period of time. In routine clinical practice (real life), the decision to choose a specific biologic needs to take into consideration that patients may often be affected by multiple comorbidities, receive concomitant medication, and necessitate treatment for a greater duration, characteristics that are profoundly different from a RCT. It is increasingly recognised that the persistence in treatment is a good surrogate of both effectiveness (efficacy in the real-life setting) and tolerability of a drug (Saad et al., 2011).

The short- and long-term benefit of adalimumab for the treatment of PsA is already documented from several clinical trials and meta-analyses (Gladman et al., 2007; Mease et al., 2009; Burmester et al., 2013). However, little evidence is available on the persistence and effectiveness of adalimumab administered as first- or second-line biologic treatment in the real-life setting.

The results from this real-life study indicate that adalimumab can be considered as a therapeutic option for the long-term treatment in PsA patients, regardless of their prior exposure to biologics or DMARDs or the presence of comorbid diseases. The majority of patients (86.6%) retained treatment with adalimumab for up to 1 year with only a slight reduction observed at 2 years (73.8% persistence), corroborating with findings from European registry studies (70–88%) for 1 year persistence rates (Heiberg et al., 2008; Saad et al., 2009; Glintborg et al., 2011; Aaltonen et al., 2017; Stober et al., 2018). However, our results showed higher rates than another real-life registry performed in Italy, with 2-year retention rate of 48% in PsA patients treated with golimumab (Manara et al., 2017). Furthermore, in that study, no difference was observed in retention rate between first- and second-line treatment in patients with rheumatoid arthritis or PsA (Manara et al., 2017). Although clinical characteristics and disease severity of patients treated in our study were similar to European registries, it is important to note that 68.6% were naïve to biologics and 55.2% were receiving concomitant DMARDs (43.2% in combination with MTX). These features may favour drug response and persistence, since evidence suggests that response to adalimumab is lower after previous TNF inhibitor (Merola et al., 2017) and concomitant MTX can improve anti-TNF drug survival (Glintborg et al., 2011), although other studies dispute the benefit of combined use of DMARDs and anti-TNF agents on drug survival (Mease et al., 2015; Aaltonen et al., 2017). In addition, we did not observe any advantage in drug persistence in patients treated with adalimumab as monotherapy compared to those receiving MTX and/or other DMARDs. Recently, a retrospective single-centre cohort study based in the UK was performed in patients with PsA who initiated anti-TNF therapy (adalimumab in 42% of cases) (Stober et al., 2018). Retention rates were similar to those observed in our study at 12 (79%) and 24 months (73%). Interestingly, the presence of metabolic syndrome and female sex were identified as predictors of lower drug persistence (Stober et al., 2018), findings that have been reported by other groups (Heiberg et al., 2008; Glintborg et al., 2011), but have not been confirmed by our study. The small number of patients with metabolic syndrome and BMI value ≥30 in our cohort might account for the lack of association between obesity and worse adalimumab performance. Why female sex emerged as a predictor of better adalimumab survival rate does not seem to have a logical explanation, although this has also been observed in psoriasis patients (Verma et al., 2018). However, published data on this topic are based on study populations of PsA patients taking any TNF inhibitors and do not investigate drugs individually. Differences in patient characteristics across studies such as age, baseline disease severity and the presence of underlying fibromyalgia may also play a role in gender related differences in drug persistence. Further studies well help to clarify this result in more detail.

High levels of CRP at baseline also predicted improved adalimumab persistence at 24 months in our hands, a finding that has also been observed previously in ankylosing spondylitis (Glintborg et al., 2010) as well as PsA patients (Glintborg et al., 2011; Aaltonen et al., 2017). High CRP levels at baseline are associated with systemic inflammation and, therefore, may help identify patients with more active disease who are more likely to benefit from adalimumab treatment than patients with less inflammatory active disease.

Given the lack of association observed in our analysis between a range of laboratory and clinical variables with persistence rate, adalimumab may be considered as a viable therapeutic option in a heterogeneous population of patients in the real-world PsA setting, without the need to restrict treatment to specific subgroups or special patient populations.

High persistence rate was paralleled with a marked improvement in arthritis measures such as TJC, SJC, DAPSA, enthesitis, and dactylitis. PASI and CRP were also significantly improved as early as 12 months and remained stable up to 2 years. We also observed a good safety profile with adalimumab in PsA patients. Of 26.5% PsA patients who discontinued treatment with adalimumab after 2 years, only 6.1% were actually due to adverse events.

## Limitations

While the main strength of the present study lies in the large sample size (N = 424) and 2-year follow-up period, subgroup analysis for some specific clinical (e.g. tender and swollen joint count, PASI and DAPSA) and laboratory measures (e.g. CRP) were hampered by missing data for some patients. The retrospective design was another limitation. However, as this study involved 16 rheumatological centres, evaluating the real-life use of adalimumab as first or second-line treatment in biologic naïve or previous biologic failure in the PsA setting, results can be generalised to the larger Italian territory.

## Conclusion

In this large real-life cohort, the use of adalimumab was found to be highly effective in PsA patients. High retention was achieved at 1 (86.6%) and 2 years (73.8%) and given the lack of association between several laboratory and clinical variables with persistence rate, adalimumab may be considered as a viable therapeutic option in a heterogeneous population of patients in the real-world PsA setting.

## Data Availability Statement

The datasets generated for this study are available upon request from the corresponding author.

## Ethics Statement

Ethics committee approval from all participating centres and written informed consent for the anonymous use of personal data were obtained from every patient, in compliance with the Italian Legislative Decree 196/2003. The patients/participants provided their written informed consent to participate in this study.

## Author Contributions

SD'A conceived and designed the study. All authors were responsible for data collection/acquisition and have critically reviewed and approved the final version of the manuscript prior to submission.

## Conflict of Interest

The authors declare that the research was conducted in the absence of any commercial or financial relationships that could be construed as a potential conflict of interest.
